# Prevalence Rates of Self-Care Behaviors and Related Factors in a Rural Hypertension Population: A Questionnaire Survey

**DOI:** 10.1155/2013/526949

**Published:** 2013-05-30

**Authors:** Huanhuan Hu, Gang Li, Takashi Arao

**Affiliations:** ^1^Lab of Exercise Epidemiology, Graduate School of Sport Sciences, Waseda University, Mikajima, Saitama 359-1192, Japan; ^2^Institute of Chronic Diseases Control and Prevention, Beijing Center for Diseases Control and Prevention, Beijing 100013, China; ^3^Lab of Exercise Epidemiology, Faculty of Sport Sciences, Waseda University, Mikajima, Saitama 359-1192, Japan

## Abstract

The objective of this study was to investigate the self-care behaviors among hypertensive patients in primary care. A cross-sectional survey, with 318 hypertensive patients, was conducted in a rural area in Beijing, China, in 2012. Participants were mainly recruited from a community health clinic and completed questionnaires assessing their self-care behaviors, including data on adherence to a prescribed medication regimen, low-salt diet intake, smoking habits, alcohol consumption, blood pressure monitoring, and physical exercise. The logistic regression model was used for the analysis of any association between self-care behaviors and age, gender, duration of hypertension, self-rated health, marital status, education level, diabetes status, or body mass index. Subjects that adhered to their medication schedule were more likely to have hypertension for a long duration (OR, 3.44; 95% CI 1.99–5.97). Older participants (OR, 1.80; 95% CI 1.08–2.99) were more likely to monitor their blood pressure. Subjects who did not partake in physical exercise were more likely to be men, although the difference between genders was not significant (OR, 0.60; 95% CI 0.36–1.01). Patients with shorter history of hypertension, younger and being males have lower self-care behaviors. Primary care providers and public health practitioner should pay more attention to patients recently diagnosed with hypertension as well as younger male patients.

## 1. Introduction 

In China, the number of patients with cardiovascular diseases is estimated to be 230 million, of whom 200 million have hypertension [1]. In addition, cardiovascular disease is responsible for a higher mortality rate among rural residents than among residents living in urban centers [1]. Approximately 57% of the total population of China lives in the countryside, and thus, the rural areas have the highest number of hypertensive patients [2]. Poor management of hypertension in rural China has become a heavy burden on public health care [1–6].

One approach that may improve blood pressure (BP) control and be feasible for the socioeconomically disadvantaged patients is patients' involvement in their own care. Self-care behaviors have been documented as one of the main determinants of hypertension control [7–10]. Despite the benefits of evidence-based hypertension self-care behaviors in improving BP, hypertensive patients generally have low compliance with the suggested self-care behaviors. A number of studies in North America and Western Europe have shown that older age, female gender, being married, and self-efficacy were predictors of self-care behavior in patients with hypertension. [11–15]. Studies on the prevalence, awareness, and treatment of hypertension in developing countries have been widely reported in recent years [16–18]. However, studies assessing what activities individuals engage in to help manage their BP, such as medication adherence, BP monitoring, and exercise practices, are scarce in developing countries [19].

Research on hypertension self-care behaviors is vital, given that it can provide information for developing policies on support for self-care, suggest what practical action can be taken, and provide ideas on how to support self-care. 

The main objectives of this study were to investigate the prevalence rates of self-care behaviors among hypertensive patients and to explore factors associated with self-care behaviors for managing hypertension.

## 2. Methods

### 2.1. Participants

Eligible participants were aged ≥35 years and had hypertension for at least 12 months. Participants who could not communicate effectively with the study personnel or provide informed consent were excluded. A total of 890 hypertensive patients were registered in the community health clinic. Physicians screened the registered patients for eligibility for this study. One hundred and forty-three patients who did not provide contact information were excluded from the study. Of the remaining 747 patients, 456 patients met the inclusion criteria and were invited to participate in this study via telephone. As some hypertensive patients may have not attended the health clinic and were not registered, we also recruited subjects through word-of-mouth and put up a poster in the community to create awareness about the study. This recruitment process was conducted for 5 weeks. 

All interviews were conducted by trained interviewers at the study site, as per an interview guide. Interviewers were familiar with all study protocols and interview techniques before entering the field. Each interview lasted for 20 minutes on an average. 

### 2.2. Instruments

The face-to-face questionnaire was structured using insights from the literature reviews and discussions with public health professionals. Questions were divided into 3 domains: sociodemographic characteristics, hypertension-related information, and self-care behaviors. Sociodemographic data included data on gender, age, educational level (≤6 and >6 years of education), annual family income (<5 and ≥5 × 10^5^ Yuan), and marital status. Hypertension-related questions included duration of hypertension, BP measure, body height, body weight, and perceived health status (very good, good, fair, poor, and very poor). Participants who reported a good or very good perceived health status were assigned a score of 1; all the others were assigned a score of 0. Six self-care behaviors were measured on the basis of the Seventh Report of the Joint National Committee on Prevention, Detection, Evaluation, and Treatment of High Blood Pressure [7]. The self-care behaviors included adherence to medication schedule, low-salt diet intake, smoking habit, alcohol consumption, regular BP measurements, and physical exercise. 

### 2.3. Anthropometric

All measurements were conducted in the morning by trained field workers as per the WHO recommendations [20]. Height was measured to the nearest 0.5 cm and weight, to the nearest 0.1 kg. Body mass index (BMI) was calculated from the weight and height. BMI (kg/m^2^) was categorized as normal weight (18.5 ≤ BMI < 24), overweight (24 ≤ BMI < 28), and obese (BMI ≥ 28) using the Chinese criteria [21].

### 2.4. Blood Pressure Measurement

BP was measured in a sitting position after at least 5 minutes of rest by using a standardized digital BP measuring machine (Omron Digital HEM-907). The second and third BP readings were averaged.

### 2.5. Adherence to Medication Regimen

The subjects' adherence to prescribed medication was tested using 5 items. Physicians were asked about the types of antihypertensive medications and doses prescribed to the participants, and the participants were asked about the actual usage of the medications at home. For example, the questions presented were “How many kinds of agents were prescribed by your physician?” and “What is the prescribed dosage for each agent per time?” The prescribed usage was compared with the actual usage at home. Participants who took their antihypertensive medications as prescribed by the physician were considered adherent; all others were considered nonadherent.

### 2.6. Other Questionnaire Parameters

Participants who reported avoiding salt intake while cooking and eating were considered to be adherent to a low-salt diet. Participants who did not smoke on a regular basis were considered to be nonsmokers. For alcohol intake, participants who reported no alcohol consumption were considered to be abstainers. For regular BP measurements, patients who reported measuring BP 2 or more times per month (at home, in the community clinical center, or in other settings) were considered to be adherent. Participants who reported performing physical exercise for 4 or more days per week were considered as adherent to the physical exercise recommendation; all others were considered non-adherent. 

### 2.7. Data Management and Statistical Analysis

Data were double-entered and cross-checked using Epi Info, version 6, statistical software. Descriptive statistics were generated with sample size, percentage, and mean. The Student's *t*-test, Chi-square test, and Fisher Exact tests were used where appropriate. The logistic regression model was used to analyze any association between self-careand age, gender, duration of hypertension, self-rated health status, marital status, education level, diabetes status, and BMI. Values were considered to be statistically significant at *P* = 0.05. All statistical analyses were performed using IBM SPSS, version 19 (SPSS Inc., Chicago, IL, USA).

### 2.8. Ethical Considerations and Treatment

Approval for this study was obtained from the Ethical Review Board of Waseda University. Written informed consent was obtained from all participants prior to data collection. Participants were aware that they could stop the interview at any time and refuse to answer questions without a reason. At the end of the study, all participants were given a small gift for their participation. 

## 3. Results

**Figure 1:**
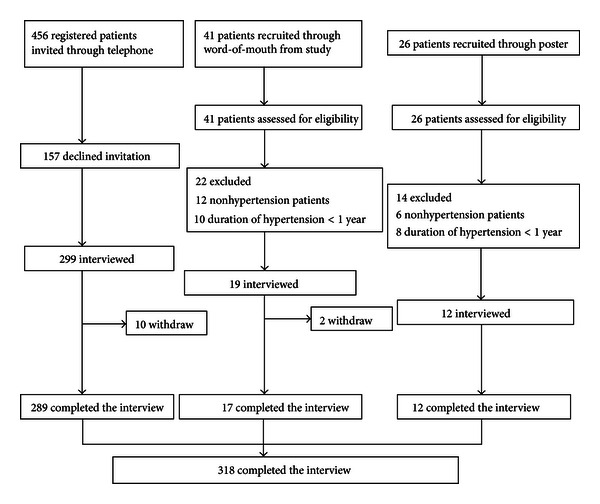
Participation rate and study cohort of survey on self-care behaviors in a rural hypertension population in Beijing, China.

A total of 523 individuals were invited to participate in the study; 456 of them were registered patients, 41 were recruited through word-of-mouth from study participants, and 26 were recruited after the poster was put up in the community (Figure 1). Among them, 318 patients (289 from registered patients, 17 from word-of-mouth, and 12 from the poster) completed the questionnaire. The overall participation rate was 60.8%.

### 3.1. Characteristics of the Sample

Demographic and hypertension-related characteristics of the sample (*n* = 318) are shown in Table 1. The average age of the participants was 62.9 (±9.8) years (range = 35–83 years). Participants reported having hypertension for an average of 8.2 (±7.1) years (range, 1–41 years). In this sample, 12.9% of the participants had their BP under control. One-fourth rated their health as good to very good. No significant differences were found for age, education level, marital status, and other characteristics between the registered patients and other participants that were recruited through the poster and word of mouth, though registered patients had a lower percentage of diabetes than other participants (18.0 versus 31.0%, *P* = 0.09) and a lower percentage of family history of hypertension (29.4 versus 44.8%, *P* = 0.08). 

### 3.2. Prevalence Rates of Hypertension Self-Care Behaviors

Approximately 81.1% of the participants reported that they avoided salt intake while cooking and eating. Approximately 79.2% of participants were nonsmokers, and 77.9% of the participants abstained from drinking any alcohol. More than half of the sample (61.3%) reported being adherent to their antihypertension medication protocols, and 51.9% of the subjects were engaging in physical exercise on most days of the week; additionally, 44.3% of the participants reported measuring BP twice or more per month either at home, at a community clinical center, or at some other setting. 

### 3.3. Factors Related to Self-Care

Using bivariate analyses, adherers and nonadherers in each of the hypertension self-care behaviors were compared using the demographic and health-relatedcharacteristics (see Table 2). Further results of multivariate analyses are shown in Table 3. Participants that maintained their medication schedule were more likely to have hypertension for a longer duration (OR 3.44, 95% CI 1.99–5.97). Older participants (≥65 years) were more likely to monitor BP (OR 1.80, 95% CI 1.08–2.99). Non-adherers of physical exercise were more likely to be men, though the difference was not significant (OR 0.60, 95% CI 0.36–1.01). Participants who were nonsmokers or adhered to a low-salt diet were more likely to be older and women as compared to the non-adherent participants. In addition, participants who abstained from alcohol were more likely to be women.

In our sample, 67 (21.1%) of the patients reported only using antihypertensive medicine when they thought their BP was high, and 56 (17.6%) patients reported not using any antihypertensive medicine. Of the 56 patients who did not use antihypertensive drugs, 25 (44.6%) of them thought their BP was not high and there was no need for treatment; 20 (35.7%) participants did not recognize the importance of medicine for BP control.

In this study, 80.2% of the participants reported not monitoring BP at home and nearly 60% of these patients did not understand or know how to measure BP. Of the patients who self-monitored at home, 68.3% used a manual BP device, and 31.7% used an automated electronic BP device. Of the participants, 258 (81.1%) reported avoiding salt intake while cooking and eating; 132 (51.2%) reported using a spoon while cooking; and 125 (48.4%) reported self-assessment of salt content while cooking. Among the non-adherers, about 66% reported that they or their family members like high salt food.

For physical exercise, 51.9% of the participants engaged in physical exercise on most days of the week. Slow walking (77.8%) was the most common physical activity in our sample. 

## 4. Discussion 

In this study, we aimed at determining the prevalence of self-care behaviors among hypertensive patients. In our sample, we found that the prevalence rates of recommended hypertension self-care activities were greater than 70% for behaviors related to smoking and alcohol consumption, and rates were much lower for self-care activities relating to medication adherence, regular blood pressure monitoring, and physical exercise. 

### 4.1. Adherence to Medication

It has been reported that antihypertensive treatment targeted to reduce systolic blood pressure produced a 38% reduction in strokes [22]. In our sample, 61.3% of the participants reported taking antihypertensive medications as prescribed, which is higher than the values reported in previous studies in China [2–4, 23]. However, the difference in study design, parameters measured, and populations often made comparisons difficult. Contrary to the reported high adherence to medication in this study, the control rate of BP was only 12.9%. There are a number of possible explanationsfor this discrepancy. One potential explanation is that patients may be likely to report desirable behavior, and the adherence to medication was probably inflated in our study. Another potential explanation is that the treatment regimens that the patients received may not have been sufficient to maintain BP in the normal range. Given the high rate (38.7%) of poor adherence to medication and that 87.1% of the subjects had uncontrolled BP, there is a critical need for enhanced treatment programs for this population. We believe that health education on the importance of adherence to medication and effective communication between patients and physicians should be focused upon for further hypertension control in this population. 

### 4.2. Access to BP Monitoring

This survey found that 37.5% of the participants monitored BP at the community health clinic or pharmacy at least twice a month. Participants who reported monitoring BP at the community health clinic or pharmacy were mostly those who lived near these facilities. Further environmental interventions providing access to BP measurement devices may play an important role in the control of BP in rural communities.

### 4.3. Awareness and Behavior Relative to Salt Reduction

Almost 80% of consumed salt is added during cooking or as a preservative of foods in rural areas of China [23, 24]. Recent surveys showed that the average salt intake is more than 10 g/day in rural areas [23, 24]. In our survey, it was difficult to assess the salt intake of the patients. Nonetheless, we found that in our sample, 81.1% of participants reported avoiding salt while cooking and eating. We noted that 51.2% of them added salt with a spoon, and 48.4% of them reported adding salt as per their own preference while cooking. These findings imply that future intervention should include education for patients on how to restrict salt intake and perhaps introduce the use of a specific salt spoon. 

### 4.4. Physical Exercise

In this sample, more than half of the participants reported participating in physical exercise. There is an ample amount of research that provides clear evidence on the positive effects of exercise on the chronic adaptation to BP. The ways by which physical activity can reduce BP may be partially explained by a decrease in systemic vascular resistance in which the autonomic nervous system and rennin-angiotensin system are most likely the underlying regulatory mechanisms [25]. However, the mechanisms related to the antihypertensive benefits of exercise are not completely understood. In addition to these physiological mechanisms that respond to exercise, loss of body weight by energy expenditure during exercise causes a reduction in BP [26]. Few people were aware of their weight problem, even though 70% of participants were overweight or obese in our sample. The patients in rural areas may not be aware that their weight status influences their BP [27]. Recent research indicates that overweight or obesity in older adults may be overlooked by health care providers, and there was a need to increase the level of communication with patients about their weight status [11, 28]. 

### 4.5. Smoking and Alcohol Consumption

In this study, the rates of smoking and alcohol consumption were both higher in men than in women. The prevalence of smoking in older patients (those aged ≥65 years) is higher than that in people aged <65 years. These findings are consistent with a study reported by Li and colleagues [29]. Multiple studies have shown that quitting smoking has proven health benefits, even at an old age [30, 31]. In our sample, nearly 70% of the subjects had less than 6 years of education. Considering that people with a lower education level have greater difficulty in quitting smoking, providing more education on the ill-effects of smoking and initiating other attempts for smoking cessation may be required for hypertensive patients. Heavy alcohol intake has also been associated with the development of hypertension [32]. Thus, heavy alcohol users should be closely evaluated for signs of hypertension. It has been observed that moderate drinking can reduce the risk for coronary artery disease [33]. However, it is still unclear whether alcohol consumption is appropriate for those with hypertension and under medication [11]. 

### 4.6. Factors Associated with Self-Care Behaviors

The results from our analyses show that older age and female gender with a longer duration of hypertension were associated with better self-care behaviors. These findings were consistent with previous research [13, 23]. It is possible that patients who have endured hypertension longer have learned more about coping with hypertension. Social and cultural factors may discourage women from smoking and alcohol intake [34]. Thus, in order to promote self-care behavior, male patients who have been recently diagnosed with hypertension should be carefully evaluated.

## 5. Strengths and Limitations

This paper is the first survey to fully describe self-care activities of hypertensive patients in China. This survey may facilitate future hypertension intervention programs, an area that is still in its infancy in China.

This study, however, has several limitations. First, this study involved use of a community-based survey and does not represent the national population. Second, as this was a cross-sectional analysis, causality could not be determined. Third, the response rate for this study was slightly lower than expected, and some of the responder characteristics might differ from the rest of the patients. Finally, the data in this study were obtained through a self-report questionnaire, and therefore, recall bias was inevitable to some extent. We could not validate participants' reported self-care activities with objective measures, especially for salt intake and medication adherence. Last, in this study, we used our own criteria to assess the adherence for each item. The results may be affected by the lack of established adherence criteria.

## 6. Conclusions

Better adherence to self-care behaviors is one effective way to control hypertension. Although more than 70% of our participants abstained from smoking and alcohol consumption, the rate of adherence to medication, regular blood pressure monitoring, and physical exercise still needs improvement. Patients with shorter history of hypertension, younger and being males have lower self-care behaviors. Primary care providers and public health practitioners should pay more attention to patients recently diagnosed with hypertension as well as younger male patients.

## Supplementary Material

A questionnaire was developed to address the goals of this study. Questions were divided into 3 domains: socio-demographic characteristics, hypertension related information, and self-care behaviors. The self-care behaviors included adherence to medication schedule, low-salt diet intake, smoking habit, alcohol consumption, regular blood pressure measurements, and physical exercise. The questionnaires will take about 20 minutes to complete.

## Figures and Tables

**Table 1:** Characteristics of respondents in a rural hypertension population in Beijing, China.

	Gender		Patients Sources		
	Male (%)	Female (%)	Registered patients (%)	Other patients (%)	Total (%)
	*N* = 90	*N* = 228	*N* = 289	*N* = 29	*N* = 318
Age					
35–64	34 (37.8)	143 (62.7)	158 (54.7)	19 (65.5)	177 (55.7)
65–83	56 (62.2)	85 (37.3)	131 (45.3)	10 (34.5)	141 (44.3)
Mean (SD)	66.1 (±10.4)	61.7 (±9.3)	63.2 (±9.8)	60.7 (±9.8)	62.9 (±9.8)
Level of education					
≤6 years	61 (67.8)	161 (70.6)	204 (70.6)	18 (62.1)	222 (69.8)
>6 years	29 (32.2)	67 (29.4)	85 (29.4)	11 (37.9)	96 (30.2)
Marital status					
Married	80 (88.9)	201 (88.2)	257 (88.9)	24 (82.8)	281 (88.4)
Others	10 (11.1)	27 (11.8)	32 (11.1)	5 (17.2)	37 (11.6)
Annual Family Income					
<50,000 yuan	86 (95.6)	223 (97.8)	281 (97.2)	28 (96.5)	309 (97.2)
≥50,000 yuan	4 (4.2)	5 (2.2)	8 (2.8)	1 (3.5)	9 (2.8)
BMI					
Normal weight (18.5 ≤ BMI < 24.0)	36 (40.0)	56 (24.5)	85 (29.4)	7 (24.1)	92 (28.9)
Overweight (24.0 ≤ BMI < 28.0)	34 (37.8)	87 (38.2)	111 (38.4)	10 (34.5)	121 (38.1)
Obese (BMI ≥ 28.0)	20 (22.2)	85 (37.3)	93 (32.2)	12 (41.4)	105 (33.0)
Self-rated health					
Good to very good	26 (28.9)	54 (23.7)	75 (26.0)	5 (17.2)	79 (24.8)
Fair to very poor	64 (71.1)	174 (76.3)	214 (74.0)	24 (82.8)	239 (75.2)
Diabetes status					
Yes	11 (12.2)	50 (21.9)	52 (18.0)	9 (31.0)	61 (19.2)
No	79 (87.8)	178 (78.1)	237 (82.0)	20 (69.0)	257 (80.8)
Family history of hypertension	19 (21.1)	79 (34.7)	85 (29.4)	13 (44.8)	98 (30.8)
Control rate of BP	14 (15.6)	27 (11.8)	37 (12.8)	4 (13.8)	41 (12.9)
Years of hypertension, Mean (SD)	8.0 (±7.3)	8.3 (±7.0)	8.2 (±6.9)	8.2 (±8.7)	8.2 (±7.1)

All values are exact numbers/percentages except where noted.

The *t*-test is used when the dependent variable is a continuous variable.

Chi-square and Fisher Exact tests were used for categorical variables.

**Table 2:** Differences between adherers and nonadherers to self-care behaviors in a rural hypertension population in Beijing, China.

	Medication adherence		Regular BP measurement		Low-salt diet		Physical exercise		Non-smoking		Alcohol abstinence	
	Adherers (*n* = 195)	Non- adherers (*n* = 123)	Adherers (*n* = 141)	Non- adherers (*n* = 177)	Adherers (*n* = 258)	Non- adherers (*n* = 60)	Adherers (*n* = 165)	Non- adherers (*n* = 153)	Adherers (*n* = 252)	Non- adherers (*n* = 66)	Adherers (*n* = 248)	Non- adherers (*n* = 70)
Age mean, SD	63.4 (9.7)	62.1 (9.8)	64.9 (8.9)	61.4 (10.2)	63.3 (9.4)	59.7 (10.9)	62.8 (9.9)	63.1 (9.7)	62.7 (9.6)	63.5 (10.7)	62.4 (9.5)	64.8 (10.5)
Education mean, SD	4.5 (3.65)	5.2 (3.6)	4.6 (3.6)	5.0 (3.7)	4.8 (3.7)	4.8 (3.3)	4.9 (3.7)	4.7 (3.6)	4.7 (3.6)	5.1 (3.8)	4.9 (3.6)	4.6 (3.9)
Duration of Hypertension Mean, SD	8.3 (6.3)	8.1 (8.2)	8.7 (7.3)	7.9 (6.9)	8.4 (6.8)	7.3 (8.4)	7.3 (6.5)	9.2 (7.5)	8.4 (6.9)	7.7 (7.8)	8.3 (7.0)	7.8 (7.2)
BMI mean, SD	26.4 (3.7)	26.4 (3.9)	26.0 (3.8)	26.7 (3.7)	26.6 (3.7)	25.7 (4.1)	26.5 (3.7)	26.3 (3.8)	26.7 (3.7)	25.1 (3.8)	26.6 (3.7)	25.8 (4.0)
Gender												
Male	55 (28.2)	35 (28.5)	41 (29.1)	49 (27.7)	64 (24.8)	26 (43.3)*	39 (23.6)	51 (33.3)	41 (16.3)	49 (74.2)*	46 (18.6)	44 (62.9)*
Female	140 (71.8)	88 (71.5)	100 (70.1)	128 (72.3)	194 (75.2)	34 (56.7)	126 (76.4)	102 (66.7)	211 (83.7)	17 (25.8)	202 (81.4)	26 (37.1)
Marital status												
Married	170 (87.2)	111 (90.2)	120 (85.1)	161 (91.0)	229 (88.8)	52 (86.7)	144 (87.3)	137 (89.5)	223 (88.5)	58 (87.9)	221 (89.1)	60 (85.7)
Others	25 (12.8)	12 (9.8)	21 (14.9)	16 (9.0)	29 (11.2)	8 (13.3)	21 (12.7)	16 (10.5)	29 (11.5)	8 (12.1)	27 (10.9)	10 (14.3)
Self-rated health												
Good to very good	47 (24.1)	32 (26.1)	36 (25.5)	43 (24.3)	66 (25.6)	13 (21.7)	42 (25.5)	37 (24.2)	62 (24.6)	17 (25.7)	57 (23.0)	22 (31.4)
Fair to very poor	148 (75.9)	91 (73.9)	105 (74.5)	134 (75.7)	192 (74.4)	47 (78.3)	123 (74.5)	116 (75.2)	190 (75.4)	49 (74.3)	191 (77.0)	48 (68.6)
Diabetes status												
No	159 (81.5)	98 (79.7)	119 (84.4)	138 (78.0)	206 (79.8)	51 (85.0)	128 (77.6)	129 (84.3)	201 (79.8)	56 (84.9)	199 (80.2)	58 (82.9)
Yes	36 (18.5)	25 (20.3)	22 (15.6)	39 (22.0)	52 (20.2)	9 (15.0)	37 (22.4)	24 (15.7)	51 (20.2)	10 (15.1)	49 (19.8)	12 (17.1)

All values are exact numbers/percentages except where noted.

The *t*-test is used when the dependent variable is a continuous variable.

Chi-square and Fisher Exact tests were used for categorical variables.

*Significant at *P* < 0.05.

**Table 3:** Associations between demographic and health characteristics and hypertension self-care behaviors in a rural hypertension population in Beijing, China.

	Medication adherence OR (95% CI)	Regular BP measurement OR (95% CI)	Low-salt diet adherence OR (95% CI)	Physical exercise OR (95% CI)	Non-smoking OR (95% CI)	Alcohol abstinence OR (95% CI)
Age						
≥65	1.11 (0.65, 1.89)	1.80 (1.08, 2.99)	3.88 (1.79, 8.48)	1.25 (0.75, 2.07)	2.29 (1.05, 4.98)	1.26 (0.65, 2.46)
<65	1.00	1.00	1.00	1.00	1.00	1.00
Gender						
Male	0.95 (0.55, 1.65)	0.89 (0.53, 1.51)	0.34 (0.17, 0.72)	0.60 (0.36, 1.01)	0.05 (0.03, 0.11)	0.13 (0.070, 0.24)
Female	1.00	1.00	1.00	1.00	1.00	1.00
Marital status						
Married	0.75 (0.35, 1.61)	0.63 (0.31, 1.28)	1.46 (0.56, 3.85)	0.80 (0.39, 1.64)	1.16 (0.40, 3.35)	1.38 (0.58, 3.28)
Others	1.00	1.00	1.00	1.00	1.00	1.00
Education						
≤6 years	1.32 (0.76, 2.29)	1.28 (0.75, 2.21)	0.51 (0.23, 1.09)	0.74 (0.44, 1.26)	0.79 (0.36, 1.71)	0.78 (0.38, 1.60)
>6 years	1.00	1.00	1.00	1.00	1.00	1.00
Self-rated health						
Good to very good	0.80 (0.46, 1.39)	0.92 (0.54, 1,56)	1.63 (0.72, 3.69)	1.11 (0.66, 1.88)	1.15 (0.54, 2.46)	0.63 (0.33, 1.21)
Fair to very poor	1.00	1.00	1.00	1.00	1.00	1.00
Diabetes status						
No	1.40 (0.76, 2.57)	1.56 (0.85, 2.86)	0.91 (0.38, 2.16)	0.64 (0.35, 1.15)	1.02 (0.41, 2.51)	1.26 (0.57, 2.78)
Yes	1.00	1.00	1.00	1.00	1.00	1.00
Duration of Hypertension						
≥3 years	3.44 (1.99, 5.97)	1.24 (0.72, 2.14)	1.92 (0.93, 3.98)	0.69 (0.40, 1.18)	1.52 (0.70, 3.28)	0.97 (0.48, 1.96)
<3 years	1.00	1.00	1.00	1.00	1.00	1.00
BMI						
BMI ≥ 28.0	0.99 (0.53, 1.87)	0.94 (0.52, 1.71)	1.36 (0.55, 3.35)	1.03 (0.57, 1.86)	1.70 (0.74, 3.90)	1.52 (0.69,3.34)
24.0 ≤ BMI < 28.0	0.75 (0.41, 1.35)	0.85 (0.48, 1.49)	0.81 (0.36, 1.80)	0.99 (0.56, 1.73)	2.33 (1.05, 5.17)	1.03 (0.51, 2.07)
18.5 ≤ BMI < 24.0	1.00	1.00	1.00	1.00	1.00	1.00

For each self-care behavior, probability modeled is adherent = “Yes”.
