# Supplementary material for: Prevalence Rates of Self-Care Behaviors and Related Factors in a Rural Hypertension Population: A Questionnaire Survey
Source: Int J Hypertens. 2013 May 30;2013:526949. doi: 10.1155/2013/526949 (PMC3683479; doi:10.1155/2013/526949)
Supplement: Supplementary file 1 — A questionnaire was developed to address the goals of this study. Questions were divided into 3 domains: socio-demographic characteristics, hypertension related information, and self-care behaviors. The self-care behaviors included adherence to medication schedule, low-salt diet intake, smoking habit, alcohol consumption, regular blood pressure measurements, and physical exercise. The questionnaires will take about 20 minutes to complete. [file 526949.f1.pdf]

Have the nature and risks of this study been explained to the patient and written informed consent obtained? ☐ Yes ☐ No If ‘No’, do not proceed.

Date informed consent was signed: ☐☐☐☐ ☐☐ ☐☐

year month day

Inclusion criteria		
All answers must be ‘YES’ for the patient to be included in the study.	Yes	No
1 Patients must be males or females >=35years of age.	☐	☐
2 Duration of hypertension >=12 months		
3 Patients must be able to communicate effectively with the study personnel.	☐	☐
4 Patients must be adequately informed of the nature and risks of the study and give written informed consent prior to screening.	☐	☐
**Exclusion criteria**		
All answers must be ‘NO’ for the patient to be included in the study.	Yes	No
1 Patients with any history of alcohol abuse, illicit drug use, significant mental illness, physical dependence to any opioid in the past year, or any history of drug use or addiction in the past year.	☐	☐
2 Women who are pregnant or breast-feeding.	☐	☐
3 Inability to complete the interview	☐	☐
4Patients who, in the opinion of the Investigator, have any other medical condition which renders the patient unable to complete the study or which would interfere with optimal participation in the study or produce significant risk to the patient.	☐	☐

Was the subject included in the study? ☐ Yes ☐ No

If ‘No’, stop interview.

Demographics

1 Age: ☐☐ years

2 Race: ☐ Han ☐ Other, please specify_________

3 Gender: ☐ Male ☐ Female

4 Education: ☐☐ years

5 Marital status: ☐ Single ☐ Married/co-habiting ☐ Divorced/separated

☐ Widowed

6 Occupation: ☐ Farmer ☐ Driver ☐ Shop keeper ☐ Worker

☐ Education/art/community service ☐ Office occupations

☐ Unemployed ☐ Retirement

7 How many of every week (7days) did you smoke on average ☐ days

8 How many of every week (7days) did you drink any alcohol on average ☐ days

9 Home income: ☐ Less than 50,000yuan ☐ 50,000yuan～

10 Medical insurance: ☐ Yes ☐ No

11 How you rate your present health: ☐ Very good ☐ Good ☐ Fair

☐ Poor ☐ Very poor

Hypertension/Diabetes history

12 Date hypertension was diagnosed: ☐☐☐☐ / ☐☐ / ☐☐

13 Readings hypertension was diagnosed: Systolic:_________ mmHg

Diastolic:__________ mmHg

14 Hypertension type: ☐ Primary ☐ Secondary ☐ Unknown

15 Family history of hypertension: ☐ Father ☐ Mother ☐ Siblings
☐ Children ☐ Spouse ☐ None

16 Did patient have Diabetes? ☐ **Yes** ☐ No If '**Yes**', complete below.

17 Diabetes Type: ☐ TypeⅠ ☐ TypeⅡ

Vital signs

18Height: ☐☐☐ cm 19 Weight: ☐☐☐ ☐ kg

20 Heart rate: ☐☐☐ bpm 21Waist circumference: ☐☐.☐ cm

Blood pressure measurement

Please take BP measurements after resting for 5 minutes and take 3 readings 2 minutes apart

Systolic Diastolic

22 Seated BP: Reading 1: ☐☐☐ / ☐☐☐ mmHg

Reading 2: ☐☐☐ / ☐☐☐ mmHg

Reading3 : ☐☐☐ / ☐☐☐ mmHg

23Frequency BP measurement: ☐ per week ☐ per month ☐ per year
☐ **rarely** ☐ **never**

24Place BP measurement: ☐ home ☐ community ☐ clinical center
☐ hospital ☐ other

25Reason for BP is **rarely** (or **never)** measured:

☐ Economic difficulty ☐ Far to get to hospital
☐ Not important for him ☐ Other, please specify___________

Home Blood pressure monitoring

26Are you taking your own measurements of BP within the home

☐ Yes ☐ **No**

27 If so, how often do you take the measurement of BP at home

Frequency use monitor: ☐ per week ☐ per month ☐ rarely

28Reason for home BP monitor: ☐

1 Advised by doctor

2 Felt unwell concerned

3 For monitoring

4 Already had access

5Other, please specify___________

29Type of monitor: ☐

1 Manual sphygmomanometers

2 Electronic sphygmomanometers

30Source of monitor: ☐

1 Pharmacies 2 Postal ordered 3 Internet 4Family members

5Friends 6 Other, please specify _________

31Reason for no home BP monitoring: ☐

1 Economic difficulty 2 Do not understand or know how

3 Not important for him 4Other,please specify_______

Adherence to medication

32Do you take anti hypertension medications now? ☐ Yes ☐ **No**

33How many kinds of medication used now ☐

1. One 2. Two 3. Three 4. More than three

34Adherence to medication:

Drug name	Drug type	Cost per month	Suggested frequency		Suggested dosage	frequency		dosage	Reasons for non-adherence
			day	week		day	week		

35Main reason for no medication ☐

1 Side effects 2 Not important for him 3 Economic difficulty

4 Far to get to hospital 5 Do not believe western medication

6 Other, please specify___________

Salt restriction

36Hypertension patients should restrict salt intake to less than 5 grams of table salt per day: ☐ Yes ☐ No ☐ Unknown

37Do you restrict table salt intake: ☐ Yes ☐ No

38How you restrict table salt intake: Salt spoon Visual assessment Other

39Main reason for salt restriction: ☐

1 Advised by doctor 2 For my own health 3 Advised by family members

4 Other, please specify_________

40How you feel your blood pressure control after salt restriction: ☐

1 Better 2 No change 3 Worse

41Main reason for no salt intake restriction: ☐

1 Do not understand or know how 2 Not important for him

3 I like high salt foods 4 Family members like high salt food

5 Other, please specify________

Leisure time physical activity

42Do you have leisure time physical activity? ☐ Yes ☐ No

43How many of the past 7 days did you do at least 30 minutes total of physical exercise? ☐ Days
